# Impact of beta-tricalcium phosphate on preventing tooth extraction-triggered bisphosphonate-related osteonecrosis of the jaw in rats

**DOI:** 10.1038/s41598-023-43315-3

**Published:** 2023-09-25

**Authors:** Naoki Funayama, Takahiro Yagyuu, Mitsuhiko Imada, Yoshihiro Ueyama, Yosuke Nakagawa, Tadaaki Kirita

**Affiliations:** Department of Oral and Maxillofacial Surgery, Nara Medical University, 840 Shijo-cho, Kashihara-shi, Nara, 634-8521 Japan

**Keywords:** Biomarkers, Diseases, Medical research, Molecular medicine, Pathogenesis

## Abstract

Antiresorptive or antiangiogenic drugs can cause medication-related osteonecrosis of the jaw that is refractory. Bisphosphonate-related osteonecrosis of the jaw (BRONJ) may be caused by procedures such as tooth extraction damage the alveolar bone, release bisphosphonates (BPs) and impede healing. This study investigated strategies for BRONJ prevention and molecular mechanisms of its onset. We assessed the effectiveness of filling extraction sockets with beta-tricalcium phosphate (β-TCP). Rats were administered zoledronic acid (ZA) 1.2 mg/kg once per week for 2 weeks, and a molar was extracted. They were randomly assigned to the β-TCP group (bone defects filled with 0.01 g of β-TCP) or control group. Tissue content measurements indicated 2.2 ng of ZA per socket in the β-TCP group and 4.9 ng in the control group, confirming BP distribution and BP adsorption by β-TCP in vivo. At 4 weeks after extraction, the β-TCP group had normal mucosal coverage without inflammation. Moreover, at 8 weeks after extraction, enhanced bone healing, socket coverage, and new bone formation were observed in the β-TCP group. Connective tissue in the extraction sockets suggested that local increases in BP concentrations may suppress the local autophagy mechanisms involved in BRONJ. Filling extraction sockets with β-TCP may prevent BRONJ.

## Introduction

Medication-related osteonecrosis of the jaw (MRONJ) is a condition in which the use of antiresorptive or antiangiogenic drugs results in an exposed jawbone^1^. It is often refractory to treatment, leading to a decreased quality of life. Bisphosphonate-related osteonecrosis of the jaw (BRONJ), a specific form of MRONJ, occurs in patients who have received bisphosphonates (BPs). Intravenous administration of BPs, such as zoledronic acid (ZA), is associated with a higher risk of developing BRONJ than oral administration. The exact cause of BRONJ is not well-understood, but it may occur when invasive procedures, such as tooth extraction, damage the alveolar bone and release BPs deposited in the bone into the invasion site, thus impeding the healing process^2,3^. Preventive measures, such as oral care before tooth extraction, prophylactic administration of antibiotics to prevent infection, and socket closure, have been suggested; however, a clear and effective preventive method is yet to be established^4–6^. Therefore, it is necessary to develop new preventive strategies for BRONJ and further investigate its pathogenesis. Hence, we focused on beta-tricalcium phosphate (β-TCP), which can adsorb BPs in vitro^7–10^. We aimed to determine whether filling the extraction socket with β-TCP can reduce the amount of free BPs in the socket and prevent BRONJ triggered by tooth extraction. Additionally, we set out to explore the molecular mechanism of the onset of BRONJ.

## Results

### Bisphosphonate distribution after alveolar bone damage and the effect of β-TCP filling

**Figure 1:**
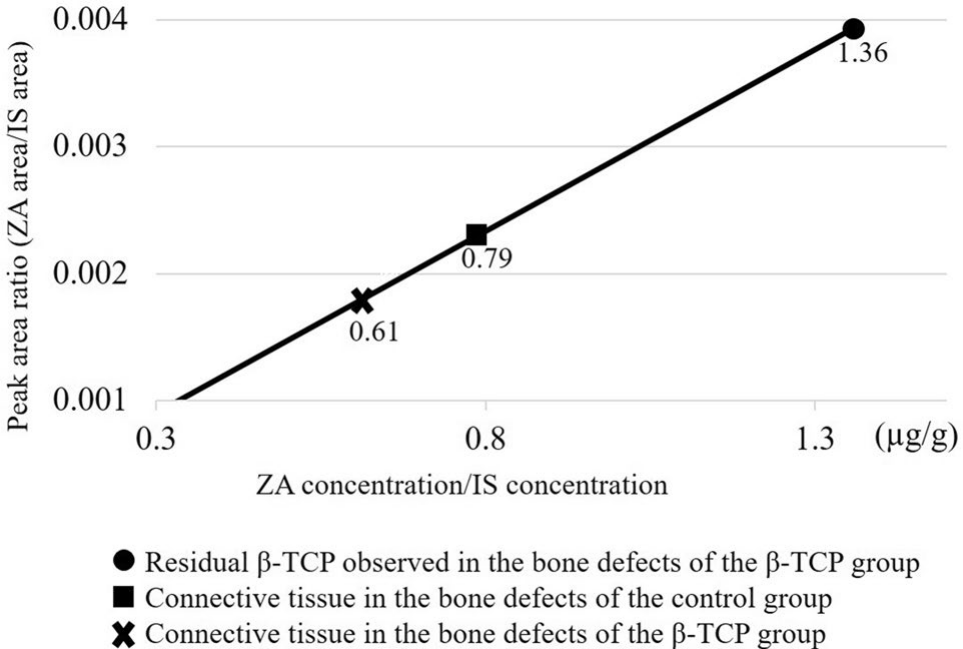
Quantification of zoledronic acid (ZA) in connective tissue and beta-tricalcium phosphate (β-TCP) within bone defects using liquid chromatography–tandem mass spectrometry. ZA was detected in the residual β-TCP (indicated by filled circle in the graph) at a concentration of 1.36 µg/g in the β-TCP group as well as in the connective tissue within the bone defects of the β-TCP group (marked by times symbol in the graph) at a concentration of 0.61 µg/g. Additionally, 0.79 µg/g of ZA was detected in the bone defects of the control group (represented by filled square in the graph) in a rat model. The internal standard (IS) solution was prepared as follows: risedronate sodium 2.5 hydrate was accurately weighed, dissolved in water to a concentration of 1 mg/mL, and diluted with physiological saline to 100 µg/mL.

To investigate the impact of invasion or β-TCP filling on BP distribution in vivo, we analyzed the amount of ZA present in β-TCP and connective tissue within extraction sockets of ZA-treated rats. The amount of connective tissue within the extraction socket was indeed reduced due to the volume occupied by the β-TCP, resulting in measurements of approximately 3.64 mg per socket in the β-TCP group, and 6.15 mg in the control group. The average amount of β-TCP collected was approximately 0.002 g, despite some slight individual differences possibly due to various factors. Remarkably, ZA was detected in residual β-TCP at 1.36 µg/g in the β-TCP group; however, ZA is not typically present in β-TCP. ZA was also found in connective tissue at 0.61 µg/g in the β-TCP group and 0.79 µg/g in the control group (Fig. 1). By calculating the amount of ZA deposited in the connective tissue per extraction socket, we found that the β-TCP group had 2.2 ng of ZA, which was less than half of the 4.9 ng found in the control group. Our findings confirmed that β-TCP can adsorb BPs in vivo, and that BPs are released from damaged alveolar bone and deposited in connective tissue around the site of invasion. Importantly, we demonstrated that the amount of BPs deposited in the connective tissue was reduced by filling the extraction socket with β-TCP because β-TCP absorbed free BP.

### Efficacy of β-TCP for preventing osteonecrosis in bisphosphonate-treated rats

#### Macroscopic evaluation of the extraction sockets

**Figure 2:**
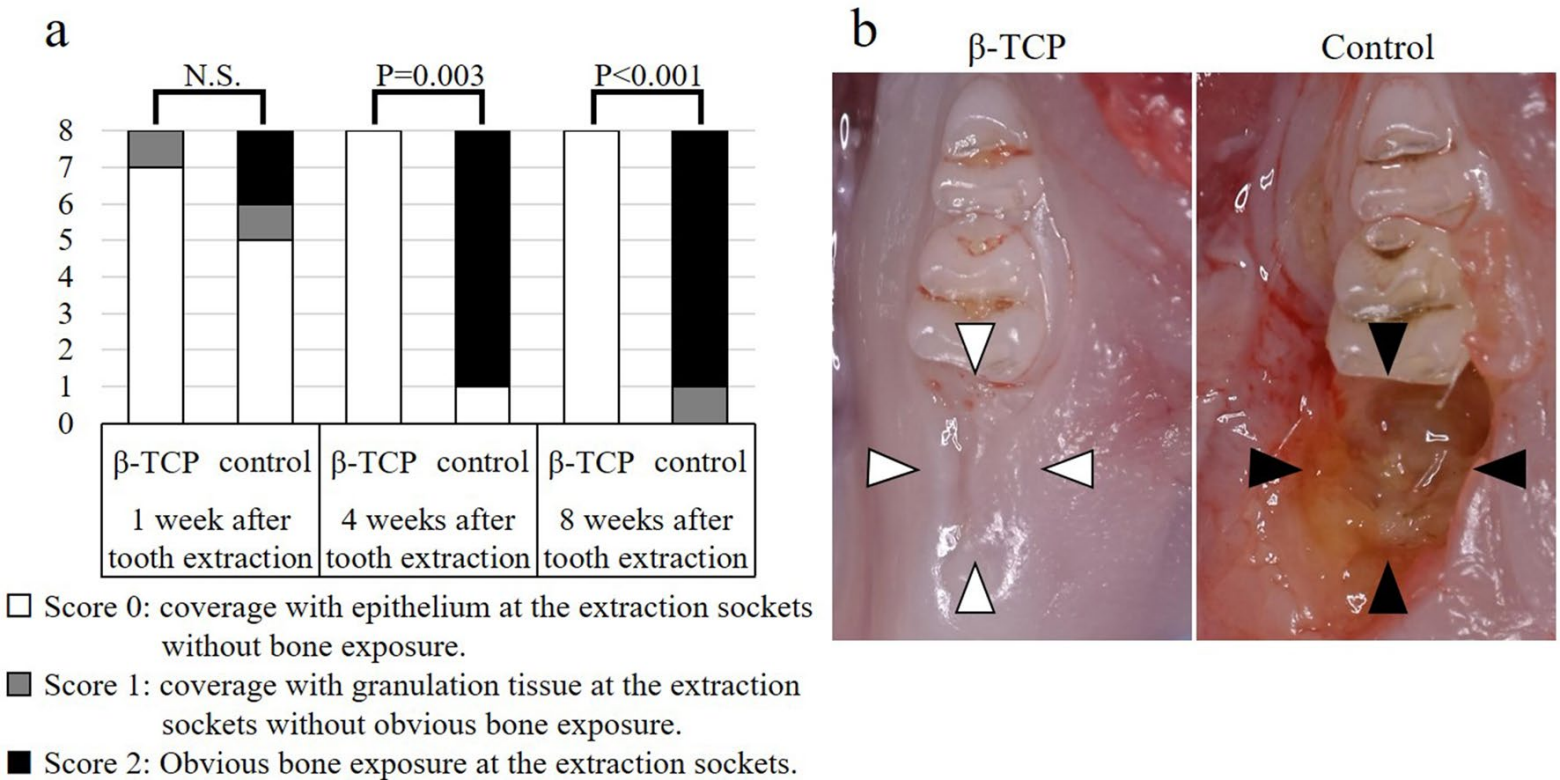
Chronological quantitative evaluation of macroscopic findings. During the chronological evaluation (**a**), all eight cases (100%) in the beta-tricalcium phosphate (β-TCP) group had a score of 0 at 4 weeks and 8 weeks after tooth extraction. However, seven of eight cases (87.5%) in the control group had a score of 2 at the same time-point. The β-TCP group had a significantly better score than the control group at the same time (P < 0.05), indicating improved mucosal healing. Representative examples of both groups (score 0 in the β-TCP group and score 2 in the control group) at 8 weeks after tooth extraction (**b**). The extraction socket of the β-TCP group (indicated by the white arrowheads) showed normal mucosal coverage without any signs of inflammation. Conversely, the extraction socket of the control group (indicated by the black arrowheads) showed bone exposure. *N.S.* not significant.

We investigated the effectiveness of β-TCP for preventing BRONJ in a rat model by macroscopically observing bone exposure in the extraction sockets at 1 week, 4 weeks, and 8 weeks after tooth extraction (Fig. 2a). There was no significant difference in the status of the extraction sockets between the two groups at 1 week after tooth extraction (P = 0.34). However, at 4 weeks after tooth extraction, all eight cases (100%) in the β-TCP group showed normal mucosal coverage without any signs of inflammation, whereas only one case (12.5%) in the control group was similarly covered, and seven of eight cases (87.5%) exhibited bone exposure that turned brown. The difference was significant (P = 0.003) and suggested that β-TCP filling effectively prevented bone exposure. At 8 weeks (Fig. 2b), normal mucosal coverage was maintained in all eight cases (100%) in the β-TCP group, and the depression of the extraction sockets became shallower compared to that at 4 weeks after tooth extraction. In contrast, seven of eight cases (87.5%) in the control group had bone exposure that expanded and resulted in discoloration of the exposed bone. Two cases had purulent drainage. Thus, β-TCP filling was found to be significantly more effective for preventing bone exposure at 8 weeks after tooth extraction (P < 0.001). Our results suggest that β-TCP filling of extraction sockets can effectively prevent BRONJ, as confirmed by macroscopic observation.

#### Micro-computed tomography evaluation of the extraction sockets

**Figure 3:**
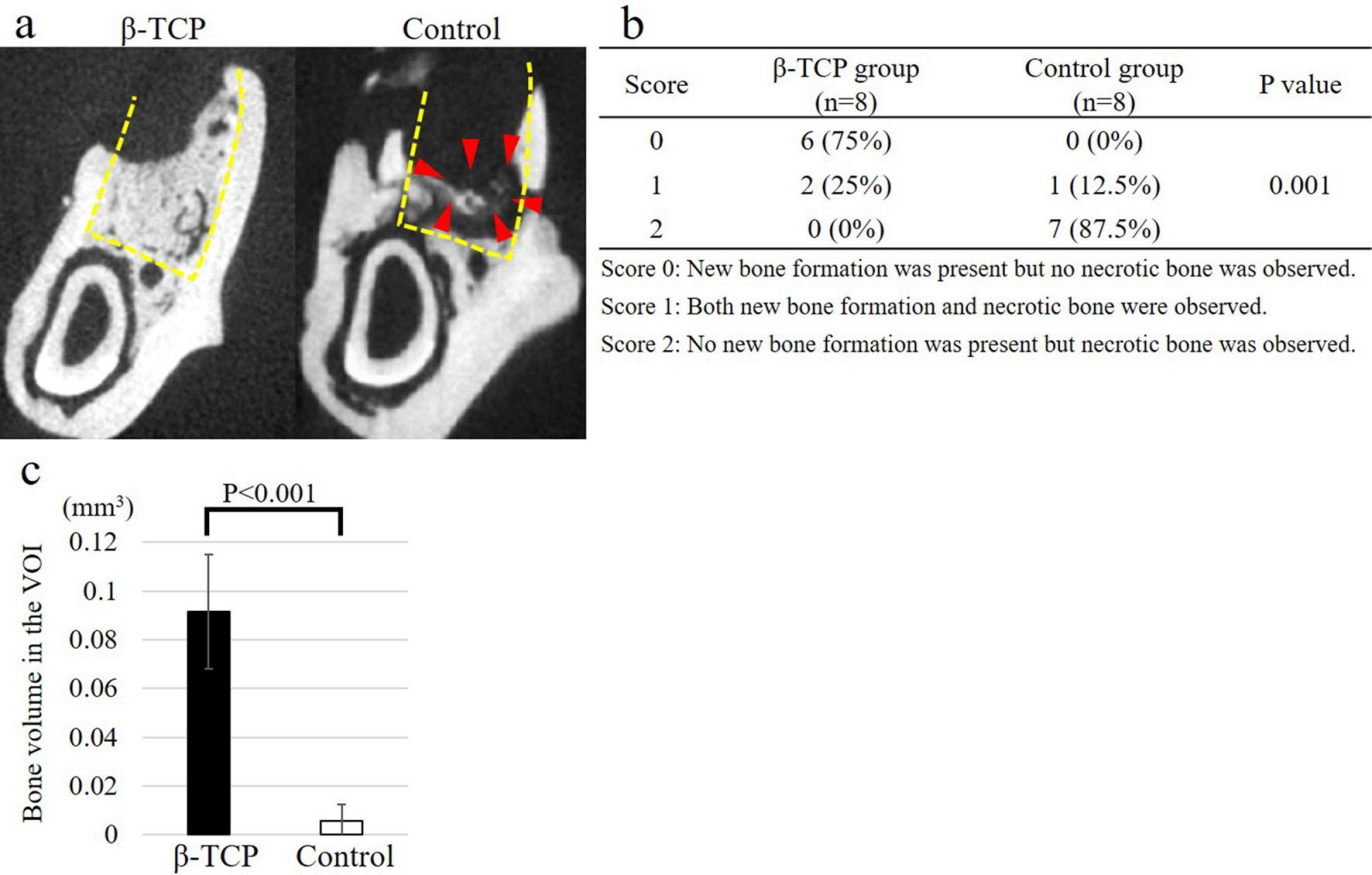
Micro-computed tomography (CT) evaluation of the extraction socket at 8 weeks after tooth extraction. In the representative micro-CT image of the extraction socket (**a**), the yellow dashed lines indicate the extraction sockets. Hard tissue with a lower CT value than the surrounding alveolar bone (suggesting new bone formation and corresponding to a score of 0 in (**b**)) was formed in the extraction socket of the beta-tricalcium phosphate (β-TCP) group. In contrast, small bone fragments separated from the surrounding alveolar bone (suggesting necrotic bone formation and corresponding to a score of 2 in (**b**)) were observed in the extraction socket of the control group, as indicated by the red arrowheads. Regarding the micro-CT evaluation findings (**b**), six of eight cases (75%) in the β-TCP group had a score of 0 and two of eight cases (25%) had a score of 1. However, in the control group, seven of eight cases (87.5%) had a score of 2 and only one case (12.5%) had a score of 1. The β-TCP group exhibited significantly lower scores than the control group (P = 0.001), indicating improved bone healing. The bone volume within the volume of interest (VOI) at 8 weeks after tooth extraction (**c**) was significantly greater in the β-TCP group than in the control group (P < 0.001). Bars represent the mean values and standard error.

We used micro-CT to evaluate the bone status of the extraction sockets of a rat model of BRONJ at 8 weeks after tooth extraction. Typically, hard tissue with a lower CT value than the surrounding alveolar bone, which suggested new bone formation (Fig. 3a, left panel), was formed in the extraction socket of the β-TCP group. In contrast, small bone fragments separated from the surrounding alveolar bone, which indicated necrotic bone formation (Fig. 3b, right panel), were observed in the extraction sockets of the control group. Then, we assessed bony healing within the extraction sockets using a three-stage scoring evaluation and compared the results between the two groups (Fig. 3b). In the β-TCP group, six of eight cases (75%) had a score of 0 and two of eight cases (25%) had a score of 1. However, in the control group, seven of eight cases (87.5%) had a score of 2 and only one case (12.5%) had a score of 1. The β-TCP group had significantly lower scores than the control group (P = 0.001), indicating improved bone healing. Furthermore, the volume of new bone formation (Fig. 3c) was significantly higher in the β-TCP group (91.6 × 10^–3^ mm^3^; standard error [SE], 8.3 × 10^–3^) than in the control group (5.4 × 10^–3^ mm^3^; SE, 2.53 × 10^–3^) (P < 0.001). Overall, these results showed enhanced bone healing in the β-TCP group and the potential of β-TCP to prevent BRONJ in the rat model.

#### Histological evaluation of the extraction sockets

**Figure 4:**
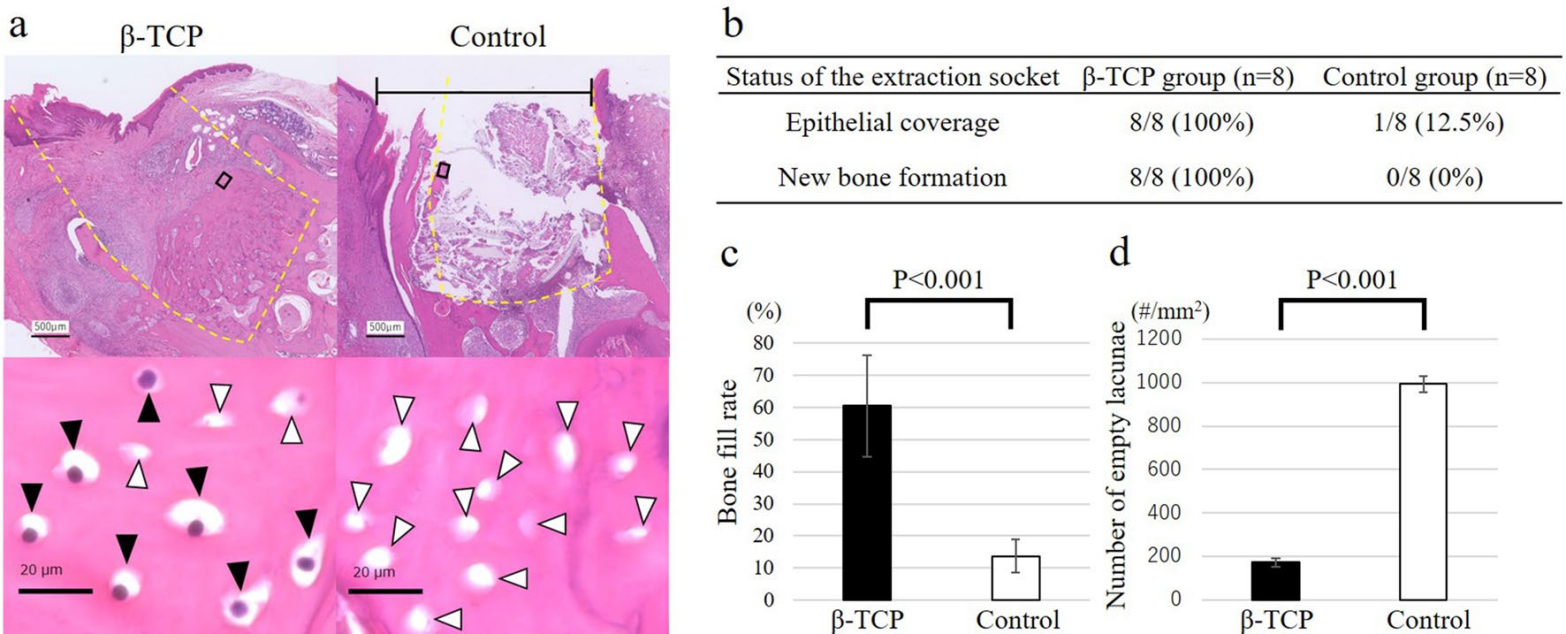
Histological evaluation of the extraction sockets at 8 weeks after tooth extraction. In a representative histological image at 8 weeks after tooth extraction (**a**), the extraction socket of the beta-tricalcium phosphate (β-TCP) group exhibited epithelial coverage and new bone formation (**a**, upper left). In the high-magnification image of the boxed area closest to the epithelium in the bone tissue within the extraction socket, numerous osteocytes with nuclei in their lacunae, indicated by black arrowheads, were observed (**a**, lower left). In contrast, the extraction sockets of the control group showed no epithelial coverage, as indicated by the black line, and displayed no new bone; only necrotic bone was displayed (**a**, upper right). In the high-magnification image of the same area, numerous empty osteocyte lacunae, indicated by white arrowheads, were observed (**a**, lower right). Yellow dashed lines mark the tooth extraction socket. (**b**) All eight cases (100%) in the β-TCP group exhibited epithelial coverage and new bone formation. However, in the control group, epithelial coverage was observed in only one of eight cases (12.5%), and new bone formation was absent in all cases. The bone-fill rate of the extraction socket in the β-TCP group was more than four-times higher than that in the control group (P < 0.001) (**c**). The number of empty osteocyte lacunae within a 200-µm area around the extraction socket in the β-TCP group was approximately one-fifth less than that in the control group (P < 0.001) (**d**). Bars represent the mean values and standard error.

We conducted a histological evaluation of the epithelial status and bone status of the extraction sockets of BP-treated rats at 8 weeks after tooth extraction. In the β-TCP group, epithelial coverage in the extraction sockets was observed along with new bone formation originating from the surrounding alveolar bone (Fig. 4a, upper left panel). The bone tissue within the extraction sockets contained numerous osteocytes with nuclei in their lacunae (Fig. 4a, lower left panel). In contrast, the control group displayed no epithelial coverage in the extraction sockets and no new bone formation (Fig. 4a, upper right panel). Only necrotic bone was observed within the extraction sockets, which exhibited numerous empty osteocyte lacunae (Fig. 4a, lower right panel). Regarding the histological findings of the epithelial status and bone status (Fig. 4b), epithelial coverage and new bone formation were observed in all eight cases (100%) in the β-TCP group. However, only one case (12.5%) in the control group exhibited epithelial coverage, whereas seven of eight cases (87.5%) lacked epithelial coverage. Furthermore, new bone formation was absent in all eight cases (100%) in the control group, with only necrotic bone present. The bone-fill rate of the extraction sockets (Fig. 4c) and the total number of empty osteocyte lacunae in the bone tissue (Fig. 4d) were quantified in each group. The β-TCP group had a significantly higher bone-fill rate (61.2%; SE, 15.6) than the control group (13.8%; SE, 5.3) (P < 0.001), whereas the number of empty osteocyte lacunae in the β-TCP group (172.1/mm^2^; SE, 18.58) was significantly lower than that in the control group (992.9/mm^2^; SE, 36.60) (P < 0.001). In summary, the histological evaluation showed that epithelial coverage and new bone formation were significantly improved in the β-TCP group, and that filling the extraction socket with β-TCP also helped prevent BRONJ pathologically.

### Verifying the modulation of protein expression by proteome and immunohistochemical analyses of bisphosphonate-treated rats that received β-TCP treatment

**Figure 5:**
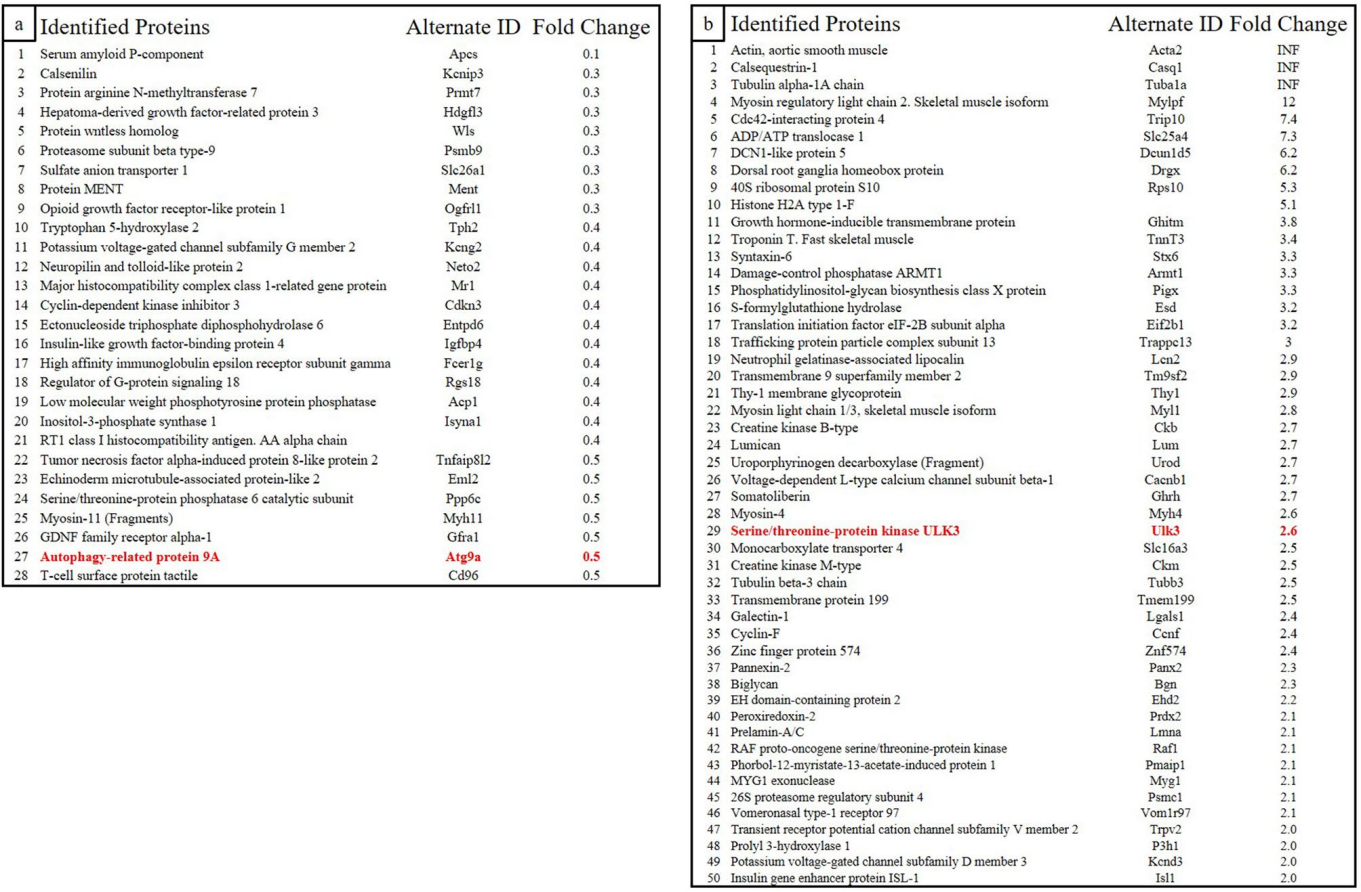
Proteins with expression modulation identified by the proteomic analysis. The expression of 5210 proteins was comprehensively searched, and 78 proteins with significant differences in expression were excerpted. In the beta-tricalcium phosphate (β-TCP) group, the expression of 28 proteins decreased to less than 50% (**a**) and the expression of 50 proteins increased more than twice (**b**). The fold change refers to the expression magnification in the β-TCP group compared to that in the control group. INF, infinity; represents proteins for which the denominator was 0 because they were not detected in the control group.

To elucidate the molecular mechanism of the onset of BRONJ, we performed a proteome analysis of the connective tissue in the extraction sockets of each group at 1 week after tooth extraction and analyzed the protein expression in connective tissue within the extraction sockets with changes in BP concentration. We comprehensively searched for the expression status of 5210 types of proteins, and a two-fold or greater modulation was observed in the expression of 78 types of proteins between both groups. The expression of 28 types of proteins decreased to less than half (Fig. 5a) and that of 50 types of proteins increased more than two-fold (Fig. 5b) in the β-TCP group.

**Figure 6:**
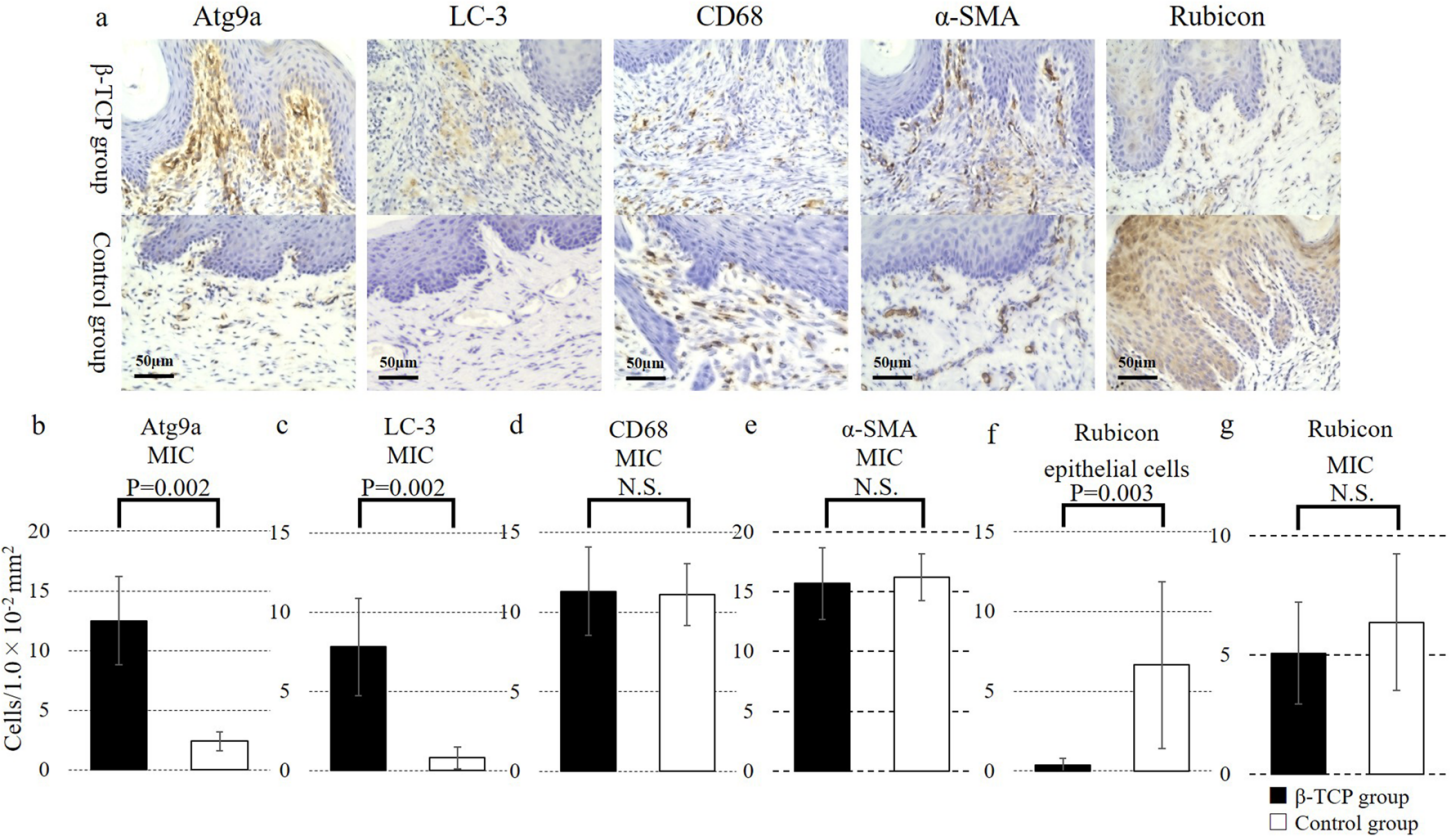
Immunohistochemical evaluation of proteins related to wound healing at 8 weeks after tooth extraction. In this representative immunohistochemical image (**a**), the upper row shows images from the beta-tricalcium phosphate (β-TCP) group and the lower row shows images from the control group for each molecule. Atg9a and LC-3 were expressed in mononuclear inflammatory cells (MICs) in the subepithelial region, and significantly more positive cells were observed in the β-TCP group (P = 0.002 and P = 0.002, respectively) (**b**,**c**). CD68 and α-SMA were expressed in MICs in the subepithelial region, but there was no significant difference in the number of positive cells between groups (**d**,**e**). Rubicon was expressed in both the stratum spinosum and MICs in the subepithelial region. The number of positive cells in the stratum spinosum (**f**) was significantly lower in the β-TCP group (P = 0.003), and the number of Rubicon-positive cells in MICs (**g**) in the subepithelial region was not significantly different between groups. Bars represent the mean values and standard error. *N.S.* not significant.

We identified autophagy-related protein 9A (Atg9a) and UNC-51 like kinase 3 (ULK3), which are autophagy-related proteins, excluding muscle and fibrous proteins, which are typical components of periodontal tissue, among the 78 potential markers. As autophagy is involved in cell remodeling and its inhibition may lead to impaired wound healing, we used immunohistochemistry to investigate the changes in the expression of the autophagy-related proteins Atg9a and LC-3 as well as the autophagy inhibitor Rubicon in the extraction sockets of BP-treated rats after filling them with β-TCP. Atg9a is a membrane protein that is essential for autophagosome formation and has a role in the early stages of autophagy. LC-3 is involved in autophagosome formation and serves as a marker for autophagic activity. Rubicon is an autophagy inhibitor that suppresses the fusion of autophagosomes and lysosomes, thus negatively regulating the autophagy process. Additionally, we performed immunohistochemical staining for CD68 and α-SMA (Fig. 6a). CD68 is a marker for macrophages (i.e., immune cells that clear debris and secrete growth factors during wound healing). α-SMA is expressed in myofibroblasts and contributes to wound contraction and extracellular matrix formation during wound healing.

According to the immunohistochemistry analysis, Atg9a and LC-3 were not expressed in epithelial cells; instead, they were expressed in mononuclear inflammatory cells (MICs) located in the subepithelial region. There were 12.5 per 1.0 × 10^–2^ mm^2^ (SE, 3.68) Atg9a-positive cells (Fig. 6b) in the β-TCP group and 2.4 per 1.0 × 10^–2^ mm^2^ (SE, 0.78) Atg9a-positive cells in the control group, indicating a significant increase in expression in the β-TCP group (P = 0.002). Similarly, there were 7.8 per 1.0 × 10^–2^ mm^2^ (SE, 3.06) LC-3-positive cells (Fig. 6c) in the β-TCP group and 0.8 per 1.0 × 10^–2^ mm^2^ (SE, 0.68) LC-3-positive cells in the control group, with a significant increase in expression observed in the β-TCP group (P = 0.002). CD68 and α-SMA were expressed in MICs in the subepithelial region, but there was no significant difference in the number of positive cells between groups (Fig. 6d, e), with P values of 0.949 for CD68 and 0.897 for α-SMA.

Rubicon was expressed in both epithelial cells and MICs in the subepithelial region. The number of Rubicon-positive cells in the stratum spinosum (Fig. 6f) was significantly lower in the β-TCP group (0.38/1.0 × 10^–2^ mm^2^; SE, 0.43) than in the control group (6.7/1.0 × 10^–2^ mm^2^; SE, 5.22) (P = 0.003). However, there was no significant difference in the number of Rubicon-positive cells in MICs between the two groups (P = 0.366) (Fig. 6g).

Thus, the immunohistochemical staining findings suggested that the modulation of local autophagy mechanisms may be involved in the onset of BRONJ, and that the local increase in BP concentration may contribute to the suppression of these mechanisms.

## Discussion

Our study aimed to examine the impact of filling the tooth extraction socket with β-TCP on reducing the amount of free BPs and preventing BRONJ in rats treated with BPs and the molecular mechanism of the onset of BRONJ. The results revealed that BPs were released and accumulated in the adjacent tissues when the alveolar bone was invaded, but β-TCP effectively reduced the amount of free BPs by adsorbing them. Reduced thrombus size and improved stability and support of the soft tissue overlying the extraction socket by filling β-TCP may also be associated with the promotion of healing; however, the results indicated that reducing the amount of BPs that are released from the alveolar bone and deposited in the adjacent tissues is crucial for preventing BRONJ. Although the amount of BPs deposited per gram of connective tissue is decreased in the β-TCP group that adsorbed free BPs, β-TCP, which has an adsorbing effect, contain a larger amount of BPs than the connective tissue, and the amount of BPs per extraction socket is increased in the β-TCP group. However, BPs adsorbed to β-TCP exhibits fewer adverse effects than free BPs^11^.

The potential underlying mechanisms of BRONJ may include the adverse effects of BPs accumulated in the adjacent tissues, such as hindered bone resorption and remodeling by osteoclasts, inhibited angiogenesis, and suppressed growth of mucosal cells. Additionally, our analysis of protein expression in the adjacent tissues suggested that local increases in BPs concentration may result in the suppression of local autophagy mechanisms. Differential trends in Atg9a expression were observed at distinct time points. While proteomic analysis at 1 week after tooth extraction indicated a decrease in the β-TCP group, immunohistochemical evaluation at 8 weeks revealed an increase. We speculate that Atg9a might be reduced at the earlier 1-week mark in the β-TCP group due to its consumption in active autophagy, contrasting with its relative accumulation in the control group where autophagy may be suppressed. By the 8-week mark, the β-TCP group might have achieved normalized autophagy along with socket healing, whereas the control group, potentially experiencing sustained autophagy suppression, might show a build-up of unconsumed Atg9a subject to negative feedback mechanisms. It must be emphasized that this interpretation, while grounded in our observations, remains speculative and warrants further detailed investigation. ULK3, which functions downstream of the mTOR complex and is located at the tip of the autophagy cascade, is a marker that indicates autophagic activity, and its increase in the β-TCP group suggests that autophagy is activated relatively compared with that in the control group. In the proteomic analysis, although the expression of Rubicon exhibited no significant difference, a slight decrease with a fold change of 0.92 was observed in the β-TCP group, mirroring the trend seen in immunohistochemical staining. This might suggest a relative suppression of autophagy in the control group. We also observed significant variations in two calcium-binding proteins, calsenilin and calsequestrin-1, in the proteomics results. Calsenilin, which was downregulated in the β-TCP group, interacts with presenilins to facilitate Ca^2+^ uptake into the endoplasmic reticulum^12^. Conversely, calsequestrin-1, which was upregulated in the β-TCP group, binds with Ca^2+^ near the endoplasmic reticulum's release channels^13^. We hypothesized that the calsenilin downregulation in the β-TCP group was due to the uninteracted presenilins in the control group. Meanwhile, the relative upregulation of calsequestrin-1 in the β-TCP group was hypothesized due to its binding with excess extracellular Ca^2+^ in the control group. This may be related to Ca^2+^ homeostasis, signal transduction, and potential autophagy suppression^14,15^. Although the association between BRONJ and autophagy may also derive from the severity of inflammation associated with the stability of soft tissues and blood clot overlying the extraction sockets, it is likely that both the direct toxic effects of BPs and indirect effects through an altered autophagy mechanism contribute to the development of BRONJ.

Two main routes, chemisorption and physisorption, have been proposed for the absorption of BPs by β-TCP^7–9,16^. Chemisorption is the process during which the nitrogen in nitrogen-containing BPs bind to the hydroxyl group on the β-TCP surface^7,8^, whereas physisorption is the process during which β-TCP loads materials into its pores through non-specific and reversible Van der Waals forces^9,16^. Chemisorption results in lesser drug adsorption than that with physisorption and provides a weaker interaction^7,17^. The adsorption rate of β-TCP for materials increases in a concentration-dependent manner, with particularly fast adsorption immediately after contact. BPs are distributed mainly on the bone surface when accumulated in bone, and it is considered that the amount of BPs released by the invasion of the bone is highest immediately after tooth extraction. In other words, BPs released from damaged alveolar bone can be efficiently adsorbed by β-TCP.

β-TCP biodegrades and is replaced by bone tissue, releasing the adsorbed substances during the biodegradation process. The BP elution profile from β-TCP is biphasic, with an initial burst release followed by a slower, protracted release^11,18–21^. The rate of BP release is influenced by factors such as pH levels, hydration status, and blood coagulation^18,22,23^. The initial burst release may be less in vivo than in vitro, and the BPs are released slowly over a period of 5 to 6 weeks until β-TCP is completely biodegraded^24^.

β-TCP has the potential to function as a sustained-release carrier of ZA, since ZA that was adsorbed in the filled β-TCP is capable of re-release into the surrounding tissues in the extraction socket^7^. Our proposed mechanism for this sustained ZA release involves the biodegradation of β-TCP and its subsequent replacement by bone tissue, during which the adsorbed substances are released. There are concerns about the occurrence of potential adverse effects when BPs are released during β-TCP biodegradation. However, in vitro studies have reported that at a concentration less than 1.0 × 10^–6^ M, BPs promoted cell proliferation in human gingival fibroblast and human keratinocyte cell lines^25^, and that local administration of low-dose (less than 1.0 × 10^–6^ M) BPs promoted bone growth^26^. It was suggested that BPs released at extremely low concentrations during biodegradation of β-TCP in vivo may promote epithelialization and bone formation at the extraction socket, potentially leading to improved wound healing. Therefore, we also consider that the extremely low concentration of ZA that was released during this process may promote epithelialization and new bone formation, potentially improving wound healing.

Our study provides three novel contributions to the field of BPs and their effects on the jaw bone. First, we provided the first in vivo evidence suggesting that BPs released from the alveolar bone during an invasive process such as tooth extraction are deposited in the surrounding connective tissue. Previous studies have proposed that the onset of BRONJ is attributable to not only the inhibition of bone remodeling caused by BPs deposited in the alveolar bone but also the various adverse effects resulting from BPs released from the alveolar bone through invasion and deposited in the surrounding connective tissue^2,3^. However, the distribution of BPs after invasion was previously unclear. Second, we demonstrated the ability of β-TCP to adsorb BPs in vivo and the change in BP distribution and a decrease in BP deposition in the surrounding tissue through its use. Previous studies have proposed using β-TCP, hydroxyapatite, and biphasic calcium phosphates to suppress the adverse effects of BPs on the oral mucosa in vitro^27,28^ and prevent BRONJ through the local use of these substances in vivo^29–32^, but they did not verify the ability of calcium phosphates to adsorb BPs in vivo or reduce the amount deposited in tissue. Third, our research suggested that altered autophagy mechanisms, as an indirect result of exposure to BPs, may have a role in the development of BRONJ. Autophagy is crucial to the maintenance of cellular balance via the removal of damaged proteins, organelles, and pathogens^33,34^. Our study is the first to suggest a potential link between BRONJ and autophagy mechanisms, which have been acknowledged in connection with various diseases, including malignant cancer, non-alcoholic fatty liver disease, myocardial infarction, and neurodegenerative diseases^33–42^.

The relevant interpretations of this study can be divided into two categories. The first pertains to the use of β-TCP as a filling material in extraction sockets of patients receiving BPs. Based on our findings and that of relevant literature^11,18–21,25,26^, we propose that filling the extraction socket with β-TCP immediately after tooth extraction may be a potential method that not only reduces the adverse effects of the release of large amounts of BPs from the alveolar bone after extraction but also enhances the healing of the extraction socket. This may be achieved through a speculated continuous release of minute amounts of BPs that was absorbed by β-TCP as it gradually degrades. However, it is important to note that these interpretations are speculative and require further investigation in future studies. This method, if proven, could provide a novel approach in preventing BRONJ by focusing on the regulation of the local concentration of BPs. Furthermore, filling the extraction socket with β-TCP after removing the necrotic bone could lead to the development of a more effective and less invasive treatment for BRONJ. The second perspective relates to the possible involvement of suppression of a local autophagy mechanism in the increased BP concentration in the extraction socket. Although it remains to be determined whether the suppression of local autophagy is a cause or result of BRONJ, the fact that autophagy suppression is implicated in the development of non-alcoholic fatty liver disease suggests that there is no contradiction in considering that autophagy suppression may be involved in the development of BRONJ. Therefore, if methods to activate local autophagy can be developed, then there is potential for the development of new preventive measures and treatments for BRONJ, with Rubicon protein as a possible target molecule.

This study had some limitations. During the experiments, the extraction socket was closed by suturing to prevent β-TCP leakage from the socket; however, in actual clinical situations, it may be difficult to close the extraction socket by suturing. This issue must be addressed to apply the method of filling the extraction socket with β-TCP in clinical practice. Wearing a splint after extraction or closing the socket by blowing fibrin glue may be effective, and we plan to consider these options in the future. Furthermore, progress was observed for only 8 weeks after extraction. It is necessary to conduct long-term follow-up and verify the usefulness of this method in preventing BRONJ.

## Conclusion

This study aimed to investigate the effects of filling tooth extraction sockets with β-TCP in BP-treated rats to prevent BRONJ. We found that β-TCP effectively adsorbed BPs released from the alveolar bone, thus decreasing their deposition in surrounding tissues and reducing the risk of BRONJ. This study also explored the possible mechanisms of BRONJ, suggesting that both direct toxic effects of BPs and indirect effects through altered autophagy mechanisms may contribute to the disease. The findings demonstrated that filling tooth extraction sockets with β-TCP immediately after extraction could be a promising preventive measure against BRONJ by controlling the local concentration of BPs and enhancing the healing of the extraction socket.

## Methods

### Rat BRONJ model and surgical procedures

All animal experiments were conducted in compliance with the protocol reviewed by the Institutional Animal Care and Use Committee of Nara Medical University and approved by the President of Nara Medical University (permit numbers: 12855, 13118, 12954). The study was conducted in accordance with the relevant ARRIVE (Animal Research: Reporting of in Vivo Experiments) guidelines and regulations.

Forty-eight male Sprague–Dawley (SD) rats (Japan SLC, Shizuoka, Japan) that were 8 weeks of age were used for this study. The rats were housed in an environment with a controlled temperature and a 12-h light–dark cycle; food and water were supplied ad libitum. The BP dosage and administration intervals used for the rat BRONJ model were based on a study by Borke et al.^43^, whereas the bone defect creation method was based on a study by Imada et al.^44^. In summary, we administrated ZA at 60 μg/kg, based on the human dose, to rats once per week for 2 weeks, extracted the left lower first molar, created standardized bone defects with a size of 2 × 3 × 2 mm, and performed suturing under general anesthesia with medetomidine hydrochloride (0.15 mg/kg), midazolam (2 mg/kg), and butorphanol tartrate (2.5 mg/kg)^45^. The rats were randomly assigned to one of the following two groups: the β-TCP group with bone defects filled with 0.01 g of β-TCP and the control group with nothing applied to the defects. Finally, the extraction sockets in both the β-TCP and control groups were securely closed with nylon sutures. To harvest the mandible, rats were sacrificed by cervical dislocation under general anesthesia. All efforts were made to minimize animal suffering.

### Bisphosphonate distribution after alveolar bone damage and the effect of β-TCP filling

We aimed to examine if the β-TCP in the extraction sockets can adsorb ZA in vivo, mirroring the in vitro results from previous studies^7–10^. The focus was on determining if this process can decrease ZA deposition in the surrounding connective tissue post-invasion. For this particular experiment, we administered ZA at 1.2 mg/kg once per week for 2 weeks. This high concentration of ZA was used to ensure measurable levels. To eliminate the possibility of ZA in the blood being adsorbed by the connective tissue or the β-TCP in the socket due to bleeding during tooth extraction, we waited for the blood concentration of ZA to decrease sufficiently. As the half-life of ZA is 146 h, we performed procedures 2 weeks after the final administration of ZA. Rats were sacrificed 1 week after tooth extraction. In the β-TCP group, we collected the connective tissue and any residual β-TCP from the bone defects together, and then manually separated all β-TCP particles from the connective tissue. For the control group, we collected the connective tissue present within the bone defects. Each tissue was weighed using an electronic balance. Since the collected tissue from the individual sockets did not reach the measurable quantity of 0.05 g, we combined the β-TCP and connective tissue from each group for the calculation of ZA content. We compared the estimated ZA contents in the connective tissue between both groups to observe the changes in ZA deposition in the tissue at identical time-points. The ZA content in each tissue was measured using liquid chromatography–tandem mass spectrometry (LC–MS/MS) (n = 2).

The LC–MS/MS measurement method included the preparation of an internal standard (IS) solution (risedronate sodium 2.5 hydrate was accurately weighed, dissolved in water to a concentration of 1 mg/mL, and diluted with physiological saline to 100 µg/mL) and standard solutions (ZA diluted to concentrations of 50, 100, 500, 1000, 5000, and 10,000 ng/mL). Calibration curve samples were created by mixing 50 µL of each standard solution and 50 µL of IS. The specimens were prepared by dissolving 50 mg of the collected tissue in 2.5 mL of 5 mol/L hydrochloric acid and mixing 200 µL of the solution with 50 µL of physiological saline and 50 µL of IS. Using the peak area ratio (zoledronic acid/IS) of the calibration curve samples, a calibration curve was created using a linear regression equation and the least-squares method. The calculated values were obtained by applying the area ratio (specimen/internal standard solution) of the specimen to the calibration curve, and the ZA content was determined per gram of connective tissue.

### Evaluation of β-TCP for the prevention of osteonecrosis in bisphosphonate-treated rats

We investigated the preventive effect of β-TCP on BRONJ. We conducted macroscopic evaluations at 1 week, 4 weeks, and 8 weeks after tooth extraction, and performed radiographic and histopathological evaluations 8 weeks after the extraction (β-TCP group: n = 8; control group: n = 8).

During the macroscopic evaluation, rats were sedated with isoflurane, and bone exposure in extraction sockets was visually assessed using a three-stage scoring system (score 0: coverage with epithelium at the extraction sockets without bone exposure; score 1: coverage with granulation tissue at the extraction sockets without obvious bone exposure; score 2: obvious bone exposure at the extraction sockets).

Evaluation using micro-computed tomography (CT) was performed. The harvested mandibles were analyzed using micro-CT (BZ-X710; Keyence, Kyoto, Japan) as described in previous studies^10,44,46^. Each extraction socket was assessed using a coronal section 1.0 mm mesial from the mesial surface of the lower second molar. Hard tissue with a lower CT value that was continuous with the surrounding alveolar bone was identified as newly formed bone, whereas small bone fragments separated from the surrounding alveolar bone were considered necrotic bone. Micro-CT findings were evaluated using a three-stage scoring system (score 0: new bone formation was present, but no necrotic bone was observed; score 1: both new bone formation and necrotic bone were observed; score 2: no new bone formation was present but necrotic bone was observed). Additionally, to quantitatively evaluate the newly formed bone, we measured the calcification areas and calculated the volume of the newly formed bone within the volume of interest (VOI), which was 0.125 mm^3^ in the area between the coronal section 1.0 mm and 1.5 mm mesial from the mesial surface of the lower second molar^44^.

A histological evaluation was also performed. For the mandibles that had already undergone micro-CT analysis, we performed formalin fixation and decalcification based on the study by Imada et al.^44^. Subsequently, thin sections (thickness, 5 µm) were cut at 1.0 mm mesial from the mesial surface of the lower second molar in the buccolingual direction and stained with hematoxylin and eosin. Using an optical microscope (BZ-X710; Keyence), we performed a histological evaluation of the epithelial status and bone status of the extraction sockets. Investigators who were blinded to the results of the micro-CT analyses and identification of the rat groups performed the histological analysis independently. Moreover, to investigate bone formation in the extraction socket and the condition of the surrounding alveolar bone, we quantified the bone-fill rate of the extraction socket and the total number of empty osteocyte lacunae in the bone tissue within a 200-µm area around the extraction socket.

### Proteomic analysis of bisphosphonate-treated rats that received β-TCP treatment

Rats were randomly assigned to one of two groups (β-TCP group: n = 9; control group: n = 7). They were sacrificed 1 week after tooth extraction, and connective tissue in the bone defects in both groups were collected and evaluated for protein expression using a proteome analysis (n = 2).

Protein solution samples were reduced and alkylated as previously described^47^. LC–MS/MS and a database search were performed according to previous studies^47–54^. Proteins detected with 99.0% probability (protein false discovery rate = 0.2%) assigned by ProteinProphet^55^ containing at least two peptides that were detected with Mascot Threshold were considered positive identifications and quantified. The two LC–MS/MS technical replicates (n = 2) from each of the two groups were combined in a scaffold. Furthermore, immunohistochemical staining was performed for proteins with significantly different expression between groups during the proteome analysis.

### Immunohistochemical analysis of bisphosphonate-treated rats that received β-TCP treatment

Atg9a, LC-3, Rubicon, α-SMA, and CD68 expression were evaluated at 8 weeks after tooth extraction by immunohistochemistry of the same specimens used for histological evaluation. For immunohistochemical staining, we selected LC-3 and Rubicon, considering their associations with various diseases and frequent use in autophagy studies. Paraffin sections with a thickness of 5 μm were deparaffinized. Antigen retrieval was performed by microwaving slides in citrate buffer (pH 6.0) for 30 min. Endogenous peroxidase activity was blocked using 0.3% hydrogen peroxide prepared in methanol for 10 min at room temperature (25 °C). After several washes with phosphate-buffered saline, sections were incubated with the following primary antibodies: anti-Atg9a (1:100 dilution; Huabio Research Inc., Boston, MA, USA) at 4 °C overnight; anti-LC-3 (1:2000 dilution; Medical & Biological Laboratories Co., Tokyo, Japan) at room temperature for 60 min; anti-α-SMA (1:500 dilution; DAKO, Kyoto, Japan) at room temperature for 30 min; anti-CD68 (1:100 dilution; Bio-Rad Laboratories, Inc., Hercules, CA, USA) at room temperature for 60 min; and anti-Rubicon (1:200 dilution; Novus Biologicals, Centennial, CO, USA) at room temperature for 60 min. Sections were washed in phosphate-buffered saline and subsequently incubated with Histofine® Simple Stain Rat MAX-PO (MULTI) (Nichirei Bioscience Inc., Tokyo, Japan) at room temperature for 30 min. We visualized the immune reactions by staining slides with 3,3-diaminobenzidine tetrahydrochloride, followed by counterstaining with Mayer’s hematoxylin. The images were captured using an all-in-one fluorescence BZ-X810 microscope (Keyence, Osaka, Japan).

Four randomly selected 100-µm × 100-µm square fields were extracted from each specimen, and the number of positive cells for each immunohistochemical staining within these fields was counted. The average number of positive cells was calculated, and the differences in the expression status between groups were compared.

### Statistical analysis

The Mann–Whitney U test was used to assess the significance of the differences in bone exposure in the extraction sockets (as determined based on macroscopic findings), volume of newly formed bone (as determined by micro-CT analysis), epithelial status and bone status of the extraction sockets, bone-fill rate of the extraction sockets, total number of empty osteocyte lacunae in the bone tissue within a 200-µm area around the extraction sockets (as determined by histological analysis), and number of positive cells (as determined by immunohistochemical staining analysis)^56,57^. During the proteomics analysis, the built-in spectral count normalization function and Student’s *t*-test were used to calculate the fold changes and P values of protein abundances between groups. Differences with P < 0.05 were considered significant for both tests (Statcel-the Useful Addin Forms on Excel-4th ed.; OMS Ltd., Saitama, Japan).

## Data Availability

The datasets generated and/or analyzed by the authors during this study are available from the corresponding author on reasonable request.
